# Bioinformatics analysis of *Brucella* vaccines and vaccine targets using VIOLIN

**DOI:** 10.1186/1745-7580-6-S1-S5

**Published:** 2010-09-27

**Authors:** Yongqun He, Zuoshuang Xiang

**Affiliations:** 1 Unit for Laboratory Animal Medicine, Department of Microbiology and Immunology, and Center for Computational Medicine and Bioinformatics, University of Michigan Medical School, Ann Arbor, MI 48109, USA

## Abstract

**Background:**

*Brucella* spp. are Gram-negative, facultative intracellular bacteria that cause brucellosis, one of the commonest zoonotic diseases found worldwide in humans and a variety of animal species. While several animal vaccines are available, there is no effective and safe vaccine for prevention of brucellosis in humans. VIOLIN (http://www.violinet.org) is a web-based vaccine database and analysis system that curates, stores, and analyzes published data of commercialized vaccines, and vaccines in clinical trials or in research. VIOLIN contains information for 454 vaccines or vaccine candidates for 73 pathogens. VIOLIN also contains many bioinformatics tools for vaccine data analysis, data integration, and vaccine target prediction. To demonstrate the applicability of VIOLIN for vaccine research, VIOLIN was used for bioinformatics analysis of existing *Brucella* vaccines and prediction of new *Brucella* vaccine targets.

**Results:**

VIOLIN contains many literature mining programs (*e.g.*, Vaxmesh) that provide in-depth analysis of *Brucella* vaccine literature. As a result of manual literature curation, VIOLIN contains information for 38 *Brucella* vaccines or vaccine candidates, 14 protective *Brucella* antigens, and 68 host response studies to *Brucella* vaccines from 97 peer-reviewed articles. These *Brucella* vaccines are classified in the Vaccine Ontology (VO) system and used for different ontological applications. The web-based VIOLIN vaccine target prediction program Vaxign was used to predict new *Brucella* vaccine targets. Vaxign identified 14 outer membrane proteins that are conserved in six virulent strains from *B. abortus*, *B. melitensis*, and *B. suis* that are pathogenic in humans. Of the 14 membrane proteins, two proteins (Omp2b and Omp31-1) are not present in *B. ovis*, a *Brucella* species that is not pathogenic in humans. *Brucella* vaccine data stored in VIOLIN were compared and analyzed using the VIOLIN query system.

**Conclusions:**

Bioinformatics curation and ontological representation of *Brucella* vaccines promotes classification and analysis of existing *Brucella* vaccines and vaccine candidates. Computational prediction of *Brucella* vaccine targets provides more candidates for rational vaccine development. The use of VIOLIN provides a general approach that can be applied for analyses of vaccines against other pathogens and infection diseases.

## Background

*Brucella* is a Gram-negative, facultative intracellular bacterium that causes brucellosis in humans and animals [1]. *Brucella* are taxonomically placed in the alpha-2 subdivision of the class Proteobacteria. Traditionally there are six species of *Brucella* based on the preferential host specificity: *B. melitensis* (goats), *B. abortus* (cattle), *B. suis* (swine), *B. canis* (dogs), *B. ovis* (sheep) and *B. neotomae* (desert mice). The first four species listed in decreasing order of severity are pathogenic to humans making brucellosis a zoonotic disease. These bacteria are also amenable for use in biological warfare and bio-terrorism. Recently, two new species *B. cetaceae* (cetacean) and *B. pinnipediae* (seal) have been described [2]. Complete genome sequences of 10 *Brucella* strains are currently available in the NCBI RefSeq database. Four genomes from *B. abortus*, *B. melitensis*, and *B. suis* have been extensively analyzed [3-6].  While animal brucellosis vaccines are commercially available, there is no effective and safe human vaccine against virulent *Brucella* infections. Extensive studies on *Brucella* have recently been concentrated on understanding the mechanisms for protective *Brucella* immunity and the development of effective human brucellosis vaccines. 

VIOLIN (http://www.violinet.org) is a web-based vaccine database and analysis system. VIOLIN contains general information on microbial pathogenesis, host ranges, and host protective immunity, as well as vaccine-specific information such as vaccine type, preparation method, genetically engineered genes, and host responses in various animal models. VIOLIN contains information about 454 vaccines and vaccine candidates for 73 pathogens. VIOLIN contains many bioinformatics tools for vaccine literature mining, vaccine data analysis and integration, and vaccine target prediction. For example, VIOLIN includes Vaxmesh and Vaxpresso programs that may be used to mine vaccine literature based on MeSH controlled vocabulary and natural language processing (NLP), respectively. Dr. Yongqun He, the founder of the VIOLIN initiated and leads community-based development of the Vaccine Ontology to support vaccine integration and automated reasoning. A web-based vaccine target prediction program Vaxign available in VIOLIN is used to predict vaccine targets based on genome sequence analysis using a reverse vaccinology strategy. 

As of May 13, 2010, more than 2,000 *Brucella* vaccine-related literature papers were searchable in PubMed, and 10 *Brucella* genomes have been published in the NCBI RefSeq database. To support *Brucella* vaccine research and development, we systematically curated from the literature existing *Brucella* vaccine information, which are stored in VIOLIN for query and further analyses. Different VIOLIN tools are also used to analyze *Brucella* vaccines and predict new vaccine targets. 

## Results

### *Brucella* vaccine literature mining in VIOLIN 

**Figure 1:**
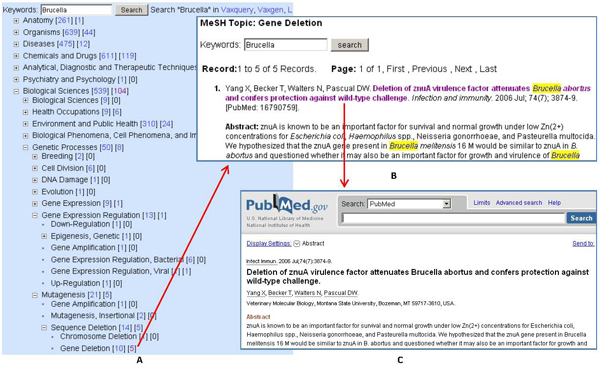
**Vaxmesh analysis of *Brucella* vaccine papers associated with the MeSH term “Gene Deletion”.** (A) Visualization of MeSH hierarchy in Vaxmesh after keyword “*Brucella*” search; (B) The two clickable numbers next to each MeSH term links to all publications with the term as a MeSH term or a major MeSH term, respectively. A click on “5” next to the MeSH term “Gene Deletion” links to another page with detailed citation information; (C) The PubMed record is accessible after a click on an article title in (B).

All *Brucella* vaccine-related articles were downloaded from PubMed and stored in VIOLIN. Information for these articles was processed and used for varying literature mining applications in VIOLIN. For example, Vaxmesh, a MeSH-based vaccine literature visualization and mining tool in VIOLIN, was used (Figure 1). The Medical Subject Headings (MeSH; http://www.nlm.nih.gov/mesh/) is the controlled vocabulary thesaurus developed by the National Library of Medicine (NLM) to index articles deposited for the MEDLINE/PubMed database. There are over 25,000 MeSH terms organized in a hierarchical fashion based on 15 top-level categories. The MeSH hierarchical structure permits literature searching at various levels of specificity. Vaxmesh provides an interactive web interface for users to locate articles using MeSH terms in a hierarchical MeSH tree structure.  Figure 1 demonstrates a MeSH hierarchy for the term “Gene Deletion”. This major MeSH term is associated with five papers in *Brucella* vaccine area (Figure 1A-1B). A click on the MeSH term links the program to another VIOLIN web page that reveals detailed information about each of the five papers. A web link to PubMed is also available (Figure 1C). According the MeSH indexing, those articles associated with *Brucella* vaccines also cover different areas such as anatomy (261 articles), physical sciences (194 articles), and geographic locations (47 articles) (Figure 1).  

Vaxperts is a new MeSH-based VIOLIN program that provides a literature-based social network of vaccine experts based on their publication records in PubMed. Vaxperts allows vaccine experts to find their co-authors and co-authors's co-authors of shared publications. This approach facilitates collaborative vaccine research and development. For example, a search for the keyword “*Brucella*” in Vaxperts resulted in the listing of 2454 authors that have contributed to at least one *Brucella* vaccine article.   

VIOLIN also contains three additional literature mining programs. These are: Vaxpresso, a natural language processing (NLP)-based vaccine literature mining program; VIOLIN Litesearch, an advanced keyword- and category-based search for vaccine literature; and Vaxlert, a literature alert program that provides periodical literature updates through Emails based on the specification of a VIOLIN user. 

### *Brucella* vaccines curated in VIOLIN 

With many literature mining programs available in VIOLIN, it is possible to make manual curation of *Brucella* vaccine information more efficient. *Brucella* vaccine curation was performed using a web-based literature mining and curation system called Limix [7,8]. Limix was developed to efficiently combine semi-automatic literature mining, manual curation, and data submission. . All curated data includes references. The curated data is published in VIOLIN and available for query only after it is critically reviewed and verified by an expert. 

VIOLIN contains 38 curated *Brucella* vaccines or vaccine candidates that have been officially licensed or proven to provide protection in an animal model (Table 1). Specifically, VIOLIN includes 20 *B. abortus* vaccines, 16 *B. melitensis* vaccines, and two *B. suis* vaccines. Among them, four *Brucella* vaccines are licensed for commercial uses in cattle, sheep, goat, and pigs. All others are research vaccines which have been demonstrated to induce protection *in vivo* against virulent *Brucella* challenges at least in some laboratory models (mostly in the mouse model). In terms of vaccine types, 1, 8, 10, and 19 vaccines are bacterial vector vaccine, DNA vaccines, subunit vaccines, and live attenuated vaccines,  respectively. 

**Table 1:** *Brucella* vaccines curated in VIOLIN and listed in VO.

#	Vaccine names	VO ID	Type	Licensed
*Brucella abortus* vaccines				

1	*B. abortus* DNA vaccine pcDNA-SOD	VO_0000018	DNA	Research
2	*B. abortus* RB51	VO_0000021	LA	Licensed
3	*B. abortus* strain 19	VO_0000022	LA	Licensed
4	*B. abortus* DNA vaccine encoding BCSP31, SOD and L7/L12	VO_0000321	DNA	Research
5	*B. abortus* subunit vaccine using L7/L12	VO_0000323	Sub	Research
6	*Brucellaabortus* bacA mutant	VO_0000347	LA	Research
7	B. recombinant SurA protein vaccine	VO_0000358	Sub	Research
8	B. recombinant DnaK protein vaccine	VO_0000373	Sub	Research
9	*B. abortus* DNA vaccine using L7/L12 and Omp16	VO_0000374	DNA	Research
10	B. *abortus* DNA vaccine encoding L7/L12 and P39	VO_0000385	DNA	Research
11	B. *abortus* with znuA deletion	VO_0000386	LA	Research
12	*B. abortus* porin-S-LPS	VO_0000403	Sub	Research
13	*B. abortus* RB51WboA	VO_0000404	LA	Research
14	Recombinant *O. anthropi* 49237SOD	VO_0000407	BT	Research
15	*B. abortus* pcDNA-BLS	VO_0000421	DNA	Research
16	Escheriosome delivery of *B. abortus* L7/L12	VO_0000423	Sub	Research
17	NPAP *Brucella* vaccine	VO_0000450	IA	Research
18	B. *abortus* strain RB51SOD	VO_0000720	LA	Research
19	B. *abortus* strain 45/20	VO_0000723	LA	Research
20	B. *abortus* S19 with P39 deletion	VO_0000826	LA	Research

*Brucella melitensis* vaccines				

21	*B. melitensis* Rev. 1 with bp26 and omp31 deletions	VO_0001171	LA	Research
22	B. *melitensis* strain VTRM1	VO_0000300	LA	Research
23	*B. melitensis* lipopolysaccharide vaccine	VO_0000311	Sub	Research
24	*B. melitensis* LPS-GBOMP noncovalent complex	VO_0000312	Sub	Research
25	*B. melitensis* DNA vaccine encoding Omp31	VO_0000325	DNA	Research
26	B. *melitensis* bp26 deletion vaccine	VO_0000338	LA	Research
27	B. *melitensis* WR201	VO_0000345	LA	Research
28	B. ovis microparticle subunit vaccine	VO_0000354	Sub	Research
29	microencapsulated *B. melitensis* mutant vaccine	VO_0000398	LA	Research
30	*B. melitensis* Bp26 and Tf vaccine	VO_0000411	Sub	Research
31	*B. melitensis* P39 recombinant protein vaccine	VO_0000412	LA	Research
32	recombinant chimera BLSOmp31	VO_0000413	Sub	Research
33	*B. melitensis* DNA vaccine encoding Omp31 boosted with Omp31	VO_0000436	DNA	Research
34	*B. melitensis* Rev. 1 with P39 deletion	VO_0000633	LA	Research
35	B. *melitensis* strain Rev. 1	VO_0000710	LA	Licensed
36	*Brucella* DNA vaccine encoding chimera BLSOmp31	VO_0001144	DNA	Research

*Brucella suis* vaccines				

37	*B. suis* strain VTRS1	VO_0000303	LA	Research
38	B. *suis* strain 2	VO_0000722	LA	Licensed

**Note:** The abbreviations LA, Sub, DNA, Con, IV, and BV represent live attenuated vaccine, subunit vaccine, DNA vaccine, conjugation vaccine, inactivated vaccine, and bacterial vector vaccine, respectively.

### Ontology representation of *Brucella* vaccines

A biomedical ontology represents the consensus-based controlled vocabularies of terms and relations which are logically formulated in such a way as to promote automated reasoning. Ontologies are able to structure complex biomedical domains and relate the myriad of data to shared understanding of biomedicine. Ontologies can be used for different purposes. The Gene Ontology (GO) is a well-known example of an ontology created for the primary purpose of providing controlled and standardized terms for naming different types of biological processes, cellular components, and molecular functions [9]. This ontology allows the common representation of attributes of gene products regardless of species of origin. Creating such ontology-based annotations is highly valuable both for querying databases and analyzing high throughput data. This has a significant impact since as of August 2010, over 2,500 peer-reviewed publications are identified through a PubMed search of “Gene Ontology”, and approximately 35,000 hits are identified through a Google Scholar search using the same keywords.  Ontologies can also be used for representation of encyclopedic knowledge, data exchange, and computational data analysis and reasoning.  

The Vaccine Ontology (VO; http://www.violinet.org/vaccineontology) is a collaborative, community-based ontology in the vaccine domain. VO can be used for vaccine data standardization, integration, and computer-assisted reasoning. VO utilizes the Basic Formal Ontology (BFO) (http://www.ifomis.org/bfo), a domain-independent ontology, as an upper level ontology. The VO was developed using the W3C standard Web Ontology Language (OWL) (http://www.w3.org/TR/owl-guide/). The latest version of VO is always available at http://purl.obolibrary.org/obo/vo.owl. In addition, VO has been listed in the OBO (Open Biomedical Ontologies) website (http://www.obofoundry.org/cgi-bin/detail.cgi?id=vaccine), and deposited in the NCBO BioPortal (http://bioportal.bioontology.org/virtual/1172). To provide a means for users to visualize the definitions and usages of VO terms and their relations, a VO Browser (http://www.violinet.org/vaccineontology/vobrowser/) was developed.       

**Figure 2:**
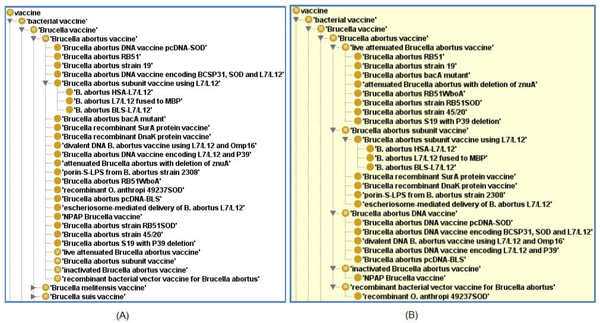
**VO hierarchy of *Brucella* vaccines.** (A) Asserted hierarchy; (B) Inferred hierarchy.

As with other vaccines, *Brucella* vaccines in VO are asserted using single inheritance based on *Brucella* species. Figure 2A demonstrates the asserted hierarchy of *B. abortus* vaccines in VO. As an OWL document, VO also supports computational inference with an OWL reasoner, such as FACT++ [10]. For example, RB51 is asserted under *Brucella abortus* vaccine (Figure 2A). Since RB51 has the qualities of ‘live’ and ‘attenuated’, it is also inferred as a ‘live attenuated *Brucella* vaccine’ using FACT++ (Figure 2B). Figure 2 provides a screenshot of *Brucella* vaccines listed in VO based on computational reasoning. 

VO has been used in many applications associated with *Brucella* vaccines. It can be used to improve PubMed searching efficiency in the vaccine domain. A user case study would be to search “live attenuated *Brucella* vaccine” in PubMed. As of April 10, 2009, a direct PubMed search of this string of keywords returned 56 papers (or PubMed hits). VO includes 13 live attenuated *Brucella* vaccines that have the qualities of ‘live’ and ‘attenuated’. When these specific *Brucella* vaccine terms were also included in a PubMed search, the number of positive paper hits in PubMed increased by more than 10-fold [11]. The combination of VO with SciMiner, a literature mining program, significantly improves PubMed searching efficiency in the general vaccine domain [12]. It was also found that the application of VO dramatically increased the performance of vaccine-induced IFN- interaction networks [13]. 

Besides vaccine hierarchy, VO can also be used to represent (or model) vaccine investigation. As demonstrated in our two recent reports, vaccine protection investigation can be represented in VO by three continuous steps: vaccination, pathogen challenge, and vaccine efficacy measurement [14,15]. A measurement of vaccine efficacy can be assessed by host survival for the pathogens (*e.g.*, Influenza virus) which kill the infected host (*e.g.*, mouse) [14] or by pathogen colony forming units (CFU), a measurement for those pathogens (*e.g.*, *Brucella*) which cannot kill infected host but exhibit diminished replication in a vaccinated host than that in unvaccinated host [15]. It is hypothesized that some parameters will play more important roles than others in determining the protection efficacy of *Brucella* vaccines. To test this hypothesis, the data for 151 groups of *Brucella* vaccine protection investigations were collected in VIOLIN from peer-reviewed literature publications and analyzed using ANOVA. Out of 16 parameters, 10 were found statistically significant (P-value <0.05) in contributing to protection based on a statistical ANOVA analysis. Examples of these parameters included vaccine strain, vaccine viability, vaccination route, vaccination dose. However, other six parameters, including IL-12 vaccine adjuvant, mouse sex, vaccination route, animal age, vaccination-challenge interval, and challenge dose, were not found statistically significant (P-value > 0.05). A careful study of this use case led to building and validating an ontology-based semantic framework to formally represent ANOVA [15]. Such an ontology-based representation of biomedical data for statistical analysis allows data consistency checking and data sharing in the Semantic Web [16].   

### Literature curation of *Brucella* protective antigens

The VIOLIN Protegen program stores protective antigens that have been verified experimentally to induce protective immunity. Protegen contains 14 protective *Brucella* antigens (Table 2). Among the 14 *Brucella* proteins, four proteins are outer membrane proteins. The other nine proteins are located in cytoplasm (5 proteins), periplasm (4 proteins), and cytoplasmic membrane (1 protein). 

**Table 2:** Vaxign-predicted vaccine targets from *B. abortus* strain 2308.

#	Locus Tag	RefSeq #	Symbol	TMH	Adhesin Prob.	Con-ed	Host Simil.	Protein Notes
**Cell Motility**								

1	BAB2_1097	YP_419224.1	FlgK	0	0.535	X		flagellar hook-associated protein FlgK
2	BAB2_1098	YP_419225.1	FlgE	0	0.749	X		flagellar hook protein FlgE
3	BAB1_0260	YP_413736.2	FlgJ	0	0.656			Flagellar protein FlgJ:Mannosyl-glycoprotein endo-beta-N-acetylglucosamidase
4	BAB1_1726	YP_415076.1		1	0.229	X		hypothetical protein

**TonB-dependent Receptor Protein: Inorganic Ion Transport and Metabolism**								

5	BAB2_0233	YP_418452.1		0	0.405	X		TonB-dependent receptor protein
6	BAB2_1150	YP_419272.1		0	0.691	X		TonB-dependent receptor protein:Pollen allergen Poa pIX/Phl pVI, C-terminal
7	BAB1_1367	YP_414742.1		0	0.655	X		TonB-dependent receptor protein

**ATP/GTP-binding Site Motif A (P-loop): Porin, Alpha proteobacteria type**								

8	BAB1_0659	YP_414101.1	Omp2a	0	0.611			Porin, alpha proteobacteria type
9	BAB1_0660	YP_414102.1	Omp2b	0	0.585	X		Porin, alpha proteobacteria type

**Cell wall/membrane/envelope Biogenesis**								

10	BAB1_0045	YP_413545.1		0	0.388	X		Bacterial surface antigen (D15)
11	BAB1_0115	YP_413611.1		0	0.793	X		outer membrane protein, putative
12	BAB1_0116	YP_413612.1		0	0.58	X		outer membrane protein, putative
13	BAB1_0707	YP_414149.1		0	0.635	X		Organic solvent tolerance protein
14	BAB1_0722	YP_414164.1	Omp25	0	0.554	X		OmpA-like transmembrane domain
15	BAB1_1176	YP_414567.1		1	0.408	X		Bacterial surface antigen (D15)
16	BAB1_1226	YP_414612.1		3	0.571	X		MotY protein: OmpA/MotB domain
17	BAB1_1302	YP_414685.1	RopB	1	0.815	X		hypothetical protein
18	BAB1_1579	YP_414943.1		1	0.669	X		OmpW family
19	BAB1_1639	YP_414995.1	Omp31-1	0	0.736	X		OmpA-like transmembrane domain
20	BAB1_1707	YP_415057.1		0	0.371	X		MotY protein: OmpA/MotB domain
21	BAB2_0314	YP_418525.1		1	0.649	X		heat resistant agglutinin 1 precursor
22	BAB1_0963	YP_414386.1		0	0.415	X		Outer membrane efflux protein

**Replication, Recombination and Repair**								

23	BAB2_0636	YP_418811.1		0	0.299	X		DNA topoisomerase I
24	BAB1_0121	YP_413617.1		0	0.162	X	X	DEAD/DEAH box helicase

**Lipid Transport and Metabolism**								

25	BAB1_0967	YP_414390.1		0	0.764			Membrane protein involved in aromatic hydrocarbon degradation

**Posttranslational Modification, Protein Turnover, Chaperones**								

26	BAB1_1944	YP_415281.1		1	0.518	X		PpiC-type peptidyl-prolyl cis-trans isomerase

**Unknown Function**								

27	BAB1_1705	YP_415055.1		0	0.594	X		TPR repeat:Molluscan rhodopsin C-terminal tail
28	BAB1_1854	YP_415198.1		0	0.759			hypothetical protein
29	BAB2_0071	YP_418316.1		0	0.492	X		hypothetical protein
30	BAB1_0069	YP_413569.1		0	0.886			hypothetical protein BAB1_0069
31	BAB1_0897	YP_414322.1		2	0.279	X		Antifreeze protein, type I
32	BAB1_0942	YP_414367.1		1	0.346	X		RNA-binding region RNP-1 (RNA recognition motif)

Abbreviations: TMH, transmembrane helixes; Adhesin Prob., Adhesin probability; Con-ed represents the conserved proteins among five other genomes from virulent *B. abortus* strain 9-941, *B. melitensis* strains 16M and ATCC 23457, and *B. suis* strains 1330 and ATCC 23445; Host Simil., similarity to human and mouse genomes.

For vaccine development against *Brucella* infections where T cell response is critical, subcellular localization is not usually an issue since a T cell response could be directed to any protein target. Our curated results confirm that protective *Brucella* antigens may occur in different subcellular locations.

### Prediction of potential *Brucella* vaccine targets

Reverse vaccinology is an emerging vaccine development approach that starts with the prediction of vaccine targets using bioinformatics screening of an entire genome of a pathogenic organism [17]. As part of VIOLIN, Vaxign is the first web-based vaccine design program that predicts vaccine targets based on reverse vaccinology [18,19]. The Vaxign computational pipeline includes the following features: subcellular localization, topology (transmembrane helices and beta barrel structure), adhesin probability, similarity to other pathogen sequences, similarity to host genome sequences (*e.g.*, human or mouse), and MHC class I and II epitope predictions.  To predict *Brucella* vaccine targets, all 10 sequenced *Brucella* genomes available in NCBI RefSeq were used for a Vaxign analysis.      

As with other intracellular pathogens, protection against *Brucella* infections requires cell-mediated immunity (CMI). Secreted pathogen proteins are likely to stimulate cytotoxic T lymphocyte (CTL) responses [20]. However, no *Brucella* protein has been found to be secreted in any *in vitro* culture in a standard culture medium. An O-sialoglycoprotein endopeptidase (Gcp; RefSeq: YP_415230.1) in *B. abortus* strain 2308 was identified by Vaxign to be a potential secreted protein. This protein is also conserved in the other virulent *B. abortus*, *B. melitensis*, and *B. suis* strains.

**Figure 3:**
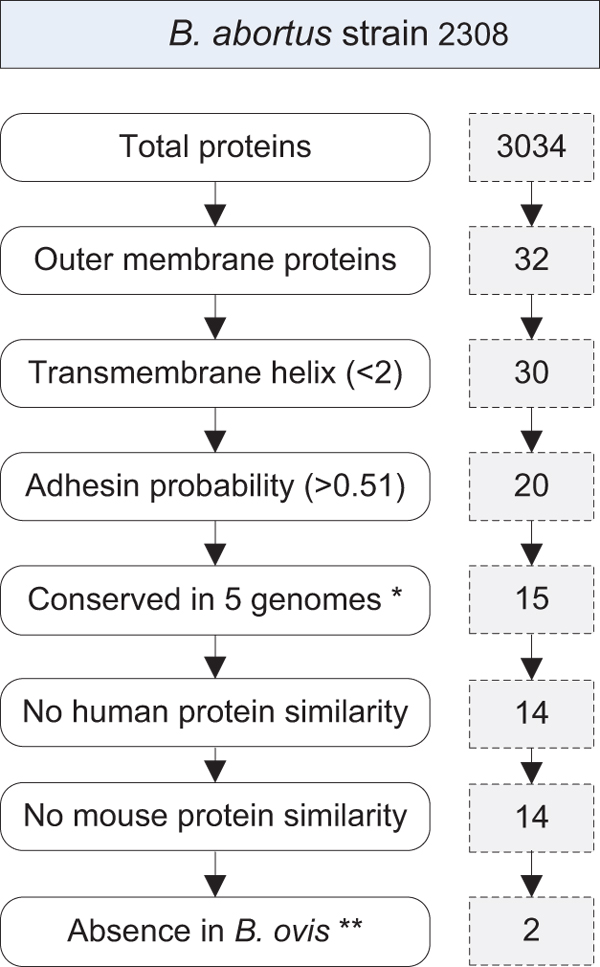
**Prediction of *Brucella* vaccine targets using Vaxign.** *, represents five genomes from virulent *B. abortus* strain 9-941, *B. melitensis* strains 16M and ATCC 23457, and *B. suis* strains 1330 and ATCC 23445. **, represents the genome from *B. ovis* strain ATCC 25840; The indicated two proteins are Omp2b (YP_414102.1) and Omp31-1 (YP_414995.1).

Vaxign was used to predict *Brucella* outer membrane proteins (OMP) as potential vaccine targets using *B. abortus* strain 2308 genome [6] as the seed genome (Figure 3). Among 3034 proteins in this genome, 32 were identified as OMPs. These OMPs from *B. abortus* strain 2308 are listed in Table 2. Some specific groups such as cell wall/membrane/envelope biogenesis and cell motility were enriched based on the COG analysis [21]. Two proteins among the 32 OMPs contain more than one transmembrane spanning region each. These two proteins are excluded for further consideration since the presence of multiple transmembrane spanning regions may make the purification of such recombinant proteins difficult [22]. Adhesins present in  microbial pathogens are essential for bacterial invasion and survival and represent possible targets for vaccine development. If only adhesins are considered, 10 out of the remaining 30 proteins have a probability < 0.51 for being an adhesin and hence were discarded. Fifteen out the remaining 20 proteins are conserved in the genomes from virulent *B. abortus* strain 9-941, *B. melitensis* strain 16M and ATCC 23457, and *B. suis* strains 1330 and ATCC 23445. Each of these strains is pathogenic to humans. One protein (BAB1_1944) has homology with human and mouse proteomes. Among these 14 predicted *Brucella* vaccine targets, Omp25 (YP_414164.1) and Omp31-1 (YP_414995.1) have been verified to be protective *Brucella* antigens [23,24]. The list of predicted targets also includes two flagellar hook proteins FlgE (YP_419225.1) and FlgK (YP_419224.1), one porin protein Omp2b (YP_414102.1), and two TonB-dependent receptor proteins BAB1_1367 and BAB2_1150. The roles of these potential proteins as protective *Brucella* antigens have not been studied. The flagellar protein FlgJ appears in *B. abortus* strains 2308 and 9-941, *B. melitensis* strain 16M, and *B. suis* strain ATCC 23445; however, FlgJ is absent from *B. suis* strain 1330 and *B. microti* strain CCM 4915. *Brucella* flagellar genes have recently been found important in *Brucella* survival *in vivo *[25]. It remains unknown whether these *Brucella* flagellar genes can be used for *Brucella* vaccine development.  

To develop a human *Brucella* vaccine, those *Brucella* proteins that exist in *Brucella* strains pathogenic to humans but are absent in *Brucella* strains that are non-pathogenic to humans would be ideal for vaccine development. Our studies have identified two proteins, Omp2b (YP_414102.1) and Omp31-1 (YP_414995.1), which are conserved in the above mentioned virulent *B. abortus*, *B. melitensis*, and *B. suis* strains that are pathogenic to humans, but absent from *B. ovis* that is non-pathogenic to humans. Omp2b and Omp31 are two major outer membrane proteins in *B. abortus *[26]. It is likely that these two proteins are critical for human-specific *Brucella* infections. If a human *Brucella* vaccine is developed, these two proteins are considered as priority antigens. A further bioinformatics analysis indicates that the porin protein Omp2b does not exist in live attenuated *B. abortus* vaccine strain 19, suggesting that Omp2b likely contributes to the attenuation of this mutant. Omp2b also exists in *B. canis* that is weakly pathogenic to humans.  However, Omp31-1 does not exist in *B. canis*. 

Vaxign identified 46 *Brucella* periplasmic proteins that are conserved in all *B. abortus*, *B. melitensis*, and *B. suis* genomes and lack sequence similarity with proteins in human or mouse genomes. The values of these proteins for vaccine development also deserve further analysis. Using the same criteria (sequence conservation and dissimilarity from human or mouse proteins), Vaxign detected approximately 1,000 cytoplasmic proteins. It is impractical to individually test this high number of proteins for vaccine development. Considering only five cytoplasmic proteins have been experimentally confirmed to be protective antigens out of 1,000 conserved cytoplasmic proteins (Table 3), it is much less likely that cytoplasmic proteins serve as  protective antigens compared to outer membrane and periplasmic proteins.

**Table 3:** *Brucella* protective antigens verified experimentally.

#	Symbol	Locus tag	Protein Description	Location	References (PMIDs)
1	BLS	CAA86936	*Brucella* lumazine synthase	Cytoplasm	11953389
2	L7/L12	BRURPL712X	Ribosomal protein L7/L12	Cytoplasm	8873388
3	P39	ABM67295	sugar-binding 39-kDa protein	Periplasm	11447155
4	Bfr	BAB2_0675	Ferritin:Bacterioferritin	Cytoplasm	11447155
5	Bp26	BMEI0536	Periplasmic immunogenic protein	Periplasm	17239499
6	DnaK	BruAb1_2100	Molecular chaperone DnaK	Cytoplasm	17686554
7	IalB	BMEI1584	Invasion protein B	Cytoplasmic membrane	17049676
8	Omp16	BAB1_1707	Outer membrane protein MotY	Outer membrane	18981242
9	Omp19	BAB1_1930	Lipoprotein Omp19	Outer membrane	18981242
10	Omp25	BMEI1249	25 kDa outer-membrane immunogenic protein precursor	Outer membrane	18981242
11	Omp31	BAB1_1639	OmpA-like transmembrane domain	Outer membrane	17014873
12	SodC	BAB2_0535	Cu/Zn superoxide dismutase	Periplasm	15039330
13	SurA	BAB1_0706	Peptidyl-prolyl cis-trans isomerase	Periplasm	17686554
14	Tig	BMEI1069	Trigger factor	Cytoplasm	17239499

Vaxign also contains an epitope prediction component that can predict MHC class I and II binding epitopes [19]. The addition of epitope prediction allows further analysis for the existence of potential *Brucella* vaccine targets.   

### Other programs in VIOLIN

**Figure 4:**
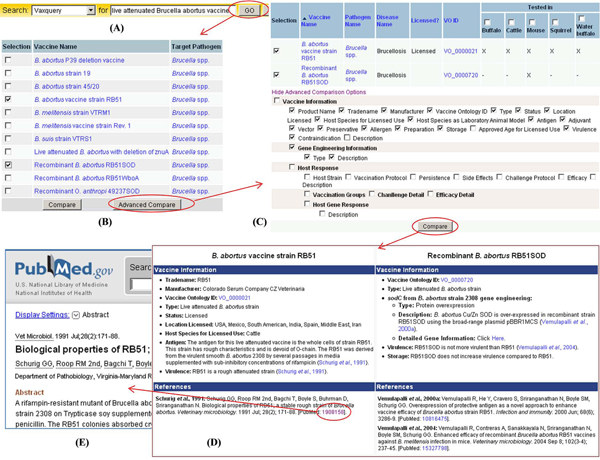
***Brucella* vaccine analyses using VIOLIN Vaxquery.** After typing the “live attenuated *Brucella abortus* vaccines” in the Vaxquery query bar (A), 11 *Brucella* vaccines were displayed (B). Two vaccines (RB51 and RB51SOD) were chosen for advanced comparison (B). After query settings were chosen (C), the results were displayed (E). All curated information has associated references which can be linked to PubMed (E).

VIOLIN provides user-friendly web interface for users to query *Brucella* vaccine data in VIOLIN. For example, Vaxquery is a user-friendly web query tool to query vaccine data (Figure 4). 

VIOLIN VBLAST is a customized BLAST sequence similarity search program. The BLAST library in VBLAST includes those vaccine-associated genes, including protective antigens, virulent factors whose mutations lead to live attenuated vaccine development, and host protective immune factors. These vaccine-associated genes can also be queried through our Vaxgen web interface. 

Two VIOLIN programs Vaxjo and Vaxvec permit analysis of vaccine adjuvants and vaccine vectors. The adjuvants used for *Brucella* vaccine development include Complete and Incomplete Freund’s Adjuvants, CpG, Cholera toxin (CT) adjuvant, Maltose binding protein (MBP). 

Additionally, VIOLIN contains the information for host responses to *Brucella* vaccines. Animal response information can be searched through VIOLIN Vaxar (http://www.violinet.org/vaxar).  Currently, annotated information for 68 host response studies of *Brucella* vaccines is available in Vaxar.  VIOLIN contains many pages that are associated with other vaccine related topics, such as vaccine conferences, manufacturers, and useful web links.

## Discussion

A large number of vaccine-related databases exist on the web. There are many government-supported vaccine databases. For example, the Centers for Disease Control and Prevention (CDC) maintain a Vaccine Information Statements (VISs) system (http://www.cdc.gov/vaccines/pubs/vis/default.htm). The Center for Biologics Evaluation and Research (CBER) under the Food and Drug Administration (FDA) regulates vaccine products and posts relevant information in their vaccine site: http://www.fda.gov/cber/vaccines.htm. There is also a Vaccine Adverse Event Reporting System (VAERS, http://vaers.hhs.gov/), co-sponsored by FDA and CDC in USA. Many agent-specific databases are also available, for example, the HIV vaccine resource (http://www3.niaid.nih.gov/research/topics/HIV/vaccines/default.htm) created by the National Institute of Allergy and Infectious Diseases (NIAID) at the National Institutes of Health (NIH). Other vaccine resources include, the Vaccine Page: http://www.vaccines.org/), the Vaccine Resource Library (PATH, http://www.path.org/vaccineresources/), and the Immunization Action Coalition (http://www.immunize.org/).  These databases primarily focus on available information concerning existing licensed vaccines and vaccine regulation. VIOLIN is unique in that it stores and analyzes research data concerning commercial vaccines and vaccines under clinical trial or in early stages of development [8]. 

The development of the Vaccine Ontology (VO) is a community effort and involves many experts in the vaccine and biomedical ontology communities [27]. With the large number of vaccine data types and publications available, VO is developed as an efficient strategy for vaccine data standardization, retrieval, and integration. VO makes it possible for computer programs to understand various vaccine types and research data associated with different vaccines. VO will also help to ensure that data is annotated in a way that ensures comparability. Therefore, VO-based software programs can be developed to support high throughput vaccine data processing and analysis. We are currently developing a VO-based literature mining and curation program that would increase the efficacy of vaccine literature mining and manual curation. The VO-based literature mining program will also relieve the burden of continuous database updating. VO will also be used to integrate all vaccine data in VIOLIN, making vaccine information exchange more efficient.

Compared to the traditional vaccine development approach that starts from a wet laboratory, reverse vaccinology begins with dry laboratory bioinformatics analysis, which makes the vaccine development more specific and efficient. Reverse vaccinology was first used by Rino Rappuoli in the development of a vaccine against serogroup B *Neisseria meningitidis* (MenB), the major cause of sepsis and meningitis in children and young adults [28]. Since then, this strategy has been applied to many other pathogens such as *Bacillus anthracis *[29], *Streptococcus pneumoniae *[30], and *Mycobacterium tuberculosis *[31]. While the criteria for vaccine prediction are known and many individual programs are available, it is still time consuming and requires expertise in these individual programs to predict vaccine targets using genome sequences. Vaxign is the first web-based automated pipeline that identifies potential vaccine targets based on the reserve vaccinology strategy [19]. Vaxign has been applied successfully to predict vaccine targets for uropathogenic *E. coli *[19]. This study demonstrated that Vaxign can predict novel *Brucella* vaccine targets. Experimental verification of many of these targets is currently under way. Vaxign also contains a program to predict immune epitopes that bind to MHC class I and II molecules in different animal species. Studies analyzing and ranking potential immune epitopes from predicted *Brucella* proteins are in progress. Promising epitopes will be tested in a wet laboratory.      

VIOLIN is also associated with other existing data resources. For example, many VIOLIN programs (*e.g.*, Vaxign and Protegen) obtain *Brucella* genome sequences and share *Brucella* gene annotations with the web-based Pathogen-host Interaction Data Integration and Analysis System (PHIDIAS, http://www.phidias.us) [32]. PHIDIAS focuses on the analysis of pathogen-host interactions. Additionally, PHIDIAS contains the *Brucella* Bioinformatics Portal, a web-based portal with a special emphasis on *Brucella* genome annotation and literature mining [7]. PHIDIAS and BBP, also developed in our group, integrate more than 20 existing data resources. The close interaction between PHIDIAS/BBP and VIOLIN makes bioinformatics analysis of *Brucella* vaccines and vaccine targets more efficient. 

VIOLIN currently includes vaccine data for 73 pathogens. The VIOLIN methods described for *Brucella* vaccine analysis in this report are generic and also feasible for vaccine studies for other pathogens. It is noted that *Brucella* is one of the most annotated pathogens among these 73 pathogens listed in VIOLIN. The vaccine information for many pathogens is not systematically annotated to the extent of *Brucella* vaccines.  More work and collaborations with the research experts in these pathogens are necessary to curate and analyze vaccines and vaccine candidates for these pathogens.      

## Conclusions

VIOLIN provides manually curated *Brucella* vaccine data and ontology representation of these vaccines using the Vaccine Ontology (VO). Many tools are developed in VIOLIN to support literature mining and data curation. Examples of data stored in the VIOLIN database include protective *Brucella* antigens and host responses induced by different *Brucella* vaccines. *Brucella* vaccine targets may be predicted using the VIOLIN Vaxign program. Various *Brucella* vaccine data can be queried using user-friendly web query programs in VIOLIN. The VIOLIN approach is generic and can be used for analyses of vaccines against other pathogens and infection diseases.  

## Methods

**Literature mining of *Brucella* vaccines using VIOLIN:** The information of all PubMed papers associated with *Brucella* vaccine and vaccination were downloaded from the PubMed web service. The literature contents were processed using VIOLIN literature mining pipelines [8]. The processed results are available for users to analyzed using individual VIOLIN literature mining programs. 

**Bioinformatics curation of *Brucella* vaccines in VIOLIN:*** Brucella* vaccine curation was performed on the VIOLIN web page using the Limix literature mining and curation system [7]. Limix allows data curators to submit data to the website, data reviewers to review and approve the submitted data, and eventual publication of high quality data. Specifically, a VIOLIN curator curates and compiles relevant information on vaccine information from peer-reviewed journals, books, and credible websites. The curated information is initially saved as a draft document and, when completed, is submitted to a MySQL database. The data submitted is initially invisible to the public and subject to critical review by an expert reviewer. Once approved, data becomes public and available for users to query. The database administrator manages users’ accounts and curation tasks. The VIOLIN database is routinely maintained by the database administrator. Published database content is periodically reviewed to ensure that new, pertinent information is captured. When new information is found, a curator and/or a domain expert will update the database content using the standard procedure described above. In addition, the VIOLIN team also periodically emails the authors of new vaccine research publications and encourages them to submit their data through the VIOLIN online submission system. VIOLIN also includes internally developed scripts to automatically update gene annotations based on updated records from existing databases (*e.g.*, NCBI Gene database). 

**VO representation of *Brucella* vaccines:** Manually curated *Brucella* vaccines are entered into VO by following the VO development standards [27]. The VO is edited by Protégé (http://protege.stanford.edu/). The FACT++ OWL reasoner [10] is used to obtain inferred *Brucella* vaccine hierarchy. 

**Vaxign prediction of *Brucella* vaccine targets:** All ten *Brucella* genomes stored in the NCBI RefSeq database were used for prediction of *Brucella* vaccine targets. The genome of *B. abortus* strains 2308 was used as a seed genome. The other genomes include five sequenced virulent strains from three main pathogenic *Brucella* species: *B. abortus* strain 9-941), *B. melitensis* strains 16M and ATCC 23457, and *B. suis* strains 1330 and ATCC 23445. These strain are pathogenic to human. The genome of *Brucella* vaccine strain S19 was also included in this study for comparison purposes. The other three *Brucella* genomes are from *B. ovis* strain ATCC 25840, *B. canis* ATCC 23365, and *B. microti* strain CCM 4915.  More *Brucella* genomes have been sequenced and available at http://www.broadinstitute.org/annotation/genome/brucella_group. Since the annotations are not yet finished and their records are not stored in the NCBI RefSeq database, these genomes were not typically used in this study. The Vaxign pipeline was executed by using the *Brucella* genomes as input data. The processed results were stored in the Vaxign database. The Vaxign web query interface was used to query and analyzed the predicted results. 

**Query of *Brucella* vaccine information in VIOLIN:** All manually curated or computational processed data can be queried through various VIOLIN web pages. Selected query functions are described in detail in the body of this manuscript. 

## List of Abbreviations

COG: The Clusters of Orthologous Groups; GO: Gene Ontology; Limix: Literature Mining and Curation System; MeSH: Medical Subject Headings; NLM: National Library of Medicine; NCBI: National Center for Biotechnology Information; NCBO: National Center for Biomedical Ontology; OBO: Open Biomedical Ontologies; OWL: Web Ontology Language; SOD: Superoxide Dismutase; VIOLIN: Vaccine Investigation and Online Information Network; VO: Vaccine Ontology; W3C: World Wide Web Consortium.

## Competing interests

The authors declare that they have no competing interests.

## Authors’ contributions

YH: *Brucella* vaccine data analysis, VIOLIN design and project manager, manuscript writing.  

ZX:  *Brucella* vaccine data analysis, VIOLIN software developer and database administrator, manuscript editing.
